# Expanding Lorentz and spectrum corrections to large volumes of reciprocal space for single-crystal time-of-flight neutron diffraction

**DOI:** 10.1107/S1600576716001369

**Published:** 2016-03-01

**Authors:** Tara M. Michels-Clark, Andrei T. Savici, Vickie E. Lynch, Xiaoping Wang, Christina M. Hoffmann

**Affiliations:** a Lawrence Berkeley National Laboratory, Berkeley, CA 94720, USA; b Oak Ridge National Laboratory, Oak Ridge, TN 37831, USA

**Keywords:** modulated diffuse scattering, local structure modeling, Lorentz and spectrum corrections, single-crystal time-of-flight neutron diffraction

## Abstract

Evidence is mounting that potentially exploitable properties of technologically and chemically interesting crystalline materials are often attributable to local structure effects, which can be observed as modulated diffuse scattering (mDS) next to Bragg diffraction (BD). BD forms a regular sparse grid of intense discrete points in reciprocal space. Traditionally, the intensity of each Bragg peak is extracted by integration of each individual reflection first, followed by application of the required corrections. In contrast, mDS is weak and covers expansive volumes of reciprocal space close to, or between, Bragg reflections. For a representative measurement of the diffuse scattering, multiple sample orientations are generally required, where many points in reciprocal space are measured multiple times and the resulting data are combined. The common post-integration data reduction method is not optimal with regard to counting statistics. A general and inclusive data processing method is needed. In this contribution, a comprehensive data analysis approach is introduced to correct and merge the full volume of scattering data in a single step, while correctly accounting for the statistical weight of the individual measurements. Development of this new approach required the exploration of a data treatment and correction protocol that includes the entire collected reciprocal space volume, using neutron time-of-flight or wavelength-resolved data collected at TOPAZ at the Spallation Neutron Source at Oak Ridge National Laboratory.

## Abstract 2

Extraction of structure factor amplitudes from total scattering (Bragg + diffuse diffraction) data for local structure modeling remains a significant challenge, especially when considering time-of-flight measurements. Individual intensity contributions carry different statistical weights, with associated distinctive effects on data summation and correction. A comprehensive, statistically improved data analysis approach to correct and scale the complete volume of reciprocal space data in one step is described.

## 1. Introduction   

Crystalline materials are primarily characterized by long-range order, which is reflected in the diffraction pattern as a series of delta functions, *i.e.* Bragg diffraction (BD). Local variations of structural subunits yield a modulated diffuse scattering (mDS) pattern. Recent examples show that a description of the local structure proved essential to interpret the structure–property relationships that underpin the material’s functionality (Aebischer *et al.*, 2006 ▸; Tu *et al.*, 2013 ▸; Welberry & Goossens, 2008 ▸; Rao *et al.*, 2013 ▸). The Bragg structure is represented by a smallest overall unit cell with translational symmetry defining the long-range order in direct space. Bragg data processing and structure refinement are essentially routine and largely automated for monochromatic X-ray diffraction. However, interpretation of the local structure from diffuse diffraction data (including measurement and appropriate application of corrections in order to prepare the data for integration) and computational modeling are much more complex (Weber & Bürgi, 2002 ▸; Bürgi *et al.*, 2005 ▸; Michels-Clark *et al.*, 2013 ▸; Welberry & Goossens, 2008 ▸). The size of the distortion in real space, *D*, affects the size of the scattering distribution in reciprocal space, 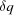, following 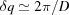 (Nield & Keen, 2001 ▸). The inverse relationship implies that the scattered intensities resulting from small local disturbances in direct space are distributed throughout reciprocal space and are generally orders of magnitude weaker in intensity than Bragg scattering. The diffuse scattering intensity distribution depends on the nature of the local structure and may be spread in one, two or three dimensions of reciprocal space (Welberry, 2004 ▸). Separating the diffuse intensities from the contributions of various independent sources of background is not trivial and needs to be addressed on a case by case basis (Bürgi *et al.*, 2005 ▸).

The majority of diffuse scattering exploration has been done for data collected on monochromatic synchrotron or home-laboratory X-ray sources. Common limitations, due to the accumulating nature of most two-dimensional area X-ray detectors, are data overflow, detector dead time and oversaturation of Bragg reflections. This necessitates separate individual data collection and data treatment of BD and mDS, and impairs consistent and simultaneous data processing and analysis. Preparation of mDS data for analysis in modeling software means that the collected raw data require complete reciprocal volume reconstructions from a series of single-wavelength wedges (= frames), which entails careful scaling schemes, removal of overexposed Bragg peaks and subtraction of background to extract representative diffuse intensities in one-, two- and three-dimensional space as lines, layers and volumes, respectively. The dimensionality of diffuse data is specific to the experiment and sample. Generally, a similar treatment is applied to BD and mDS in neutron diffraction.

Here we introduce a comprehensive protocol of combined BD and mDS data processing, equally treating the full scattering volume of reciprocal space. Data treatment and analysis are explored for time-of-flight (TOF) Laue neutron scattering patterns collected on the SNS TOPAZ single-crystal diffractometer. The instrument is equipped with state-of-the-art area detectors, with continuous readout of individual neutron events. This circumvents limitations of data overflow and oversaturation of Bragg reflections, eliminating the necessity of a separate weighting scheme for the diffuse intensities. The total scattering (Bragg + Diffuse) data of single-crystal samples are collected in volumes of reciprocal space. Every neutron is counted as an event and saved. Since event data are not accumulated into predefined bins, histograms can be defined dynamically as part of the data processing protocol.

The underlying premise of conventional data processing is based on the assumption that each Bragg peak is approximated as diffraction at one wavelength, resulting in discrete intensity points in reciprocal space. According to Bragg’s law, this produces a sparse grid leaving most of reciprocal space to background. As this only yields a minute reciprocal space volume of interest for analysis, and computing resources traditionally have been limited, it is generally sufficient to integrate the raw Bragg intensities first, and then apply scattering symmetry, instrument and wavelength dependent corrections only on the integrated Bragg intensities. The Bragg peaks are saved individually. However, in the case of diffuse data, which are distributed over considerably larger volumes of reciprocal space, a single-point correction is not applicable. Moreover, the sparse grid of BD needs to be transformed into a fine grid that covers the mDS volume, with adequate resolution to account for variations in scattering geometry and in wavelength, for the case of time-of-flight Laue data. Both requirements (dense grid and corrections) can be met simultaneously by processing the volume of reciprocal space as a whole in momentum space. Because the Bragg and modulated diffuse scattering are system dependent, the appropriate grid size needs to be adjustable for every case. Therefore, event based collection, without predefined histograms, is the ideal data acquisition mode.

In this work, data processing is examined theoretically, expanding the correction protocol and simultaneously taking the wavelength variations of TOF into account, to normalize a complete data set. Original and new data analysis procedures are compared for both Bragg and diffuse scattering for a single crystal of Er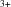 doped β-NaLaF_4_ (hexagonal phase), from a family of light emitting sodium lanthanide tetrafluorides. Information about the sample is presented in §2.1. The current data processing approach to Bragg peaks is shown in §2.2. The proposed revised protocol is described theoretically in §2.3, while an example of implementation is shown in §2.4. The results are discussed in §3.

## 2. Materials and methods   

### 2.1. Example material description: technically exploitable β-NaLaF_4_   

Several of the rare earth doped sodium lanthanide tetrafluoride compounds are efficient light up-conversion phosphors. They have been studied for their ability to act as luminescence host matrices (Haase & Schafer, 2011 ▸), a function based on efficient luminescent sites, which are generally triggered by locally asymmetric environments. The average (Bragg) crystal structure determined by X-ray diffraction (Burns, 1965 ▸) showed high symmetry and revealed no obvious link to the material properties. However, spectroscopic investigations indicated that the average structure displays an idealized high symmetry for the La atoms located at the suspect center positions (Aebischer *et al.*, 2006 ▸; Tu *et al.*, 2013 ▸). Subsequent investigation of the local structure arrangement through analysis of the associated diffuse diffraction data supported the spectroscopic findings. This shows that complementary information provided by mDS is needed to allow insight on the true structure–property relationship (Auzel, 2004 ▸; Krämer *et al.*, 2004 ▸; Aebischer *et al.*, 2005 ▸, 2006 ▸; Tu *et al.*, 2013 ▸), and understanding of the local structure is critical for targeted development and improvement of materials, in this case up-conversion properties.

Neutron diffraction provides complementary data to X-ray diffraction, owing to differences in scattering strength for the elements present. It is a mechanism to verify and enhance the description of the local structure derived from the diffuse X-ray data. Aebischer *et al.* (2006 ▸) reported a qualitative estimate of a diffuse X-ray pattern, which is dominated by La^3+^ (57 electrons) relative to F and Na (with only nine and 11 electrons, respectively). The combination of heavy and light elements present in β-NaLaF_4_ makes neutron diffraction a particularly useful technique as Na and F have more favorable coherent scattering lengths of 3.63 fm (Na) and 5.654 fm (F) compared with 8.24 fm for La (Sears, 1992 ▸).

A high-flux neutron beam is required for quantitative diffuse scattering studies. The TOPAZ single-crystal diffractometer at the SNS was used for data collection on β-NaLaF_4_. Subsequently, the new processing protocol for neutron elastic scattering data was applied and a quantitative local structure model was simulated using *ZODS*, computational modeling software in development (Chodkiewicz *et al.*, 2010 ▸). The methodology described may be applied in general to neutron diffraction data measured in TOF event mode.

### 2.2. Standard neutron scattering experiment   

Niobium doped vanadium is a purely incoherent scatterer and therefore isotropic by definition, providing a mechanism to correct for incident flux in momentum space. Diffraction data for the incoherent scattering were collected for 48 h from a spherical (*r* = 0.1375 cm) niobium doped vanadium sample. A background measured with an empty instrument was collected for a similar time, scaled to the same incident flux as the vanadium data and then subtracted. A spherical absorption correction was applied to the niobium doped vanadium data after background removal. The resulting data set was used for detector efficiency calculation and for calculating the wavelength dependent incident flux.

A single crystal of β-NaLaF_4_ (approximately one cubic millimetre) was selected from a batch of crystals grown using the Bridgeman technique by collaborators at the University of Bern (Bürgi, 2011 ▸). The crystal was mounted on an aluminium pin using Super Glue (cyanoacrylate) and placed on the goniometer equipped with a 100 K nitrogen cryostream (Cryostream 700 Plus). Neutron event data were collected using a pulsed neutron beam with a wavelength band of 0.5–3.5 Å, at 60 Hz. The first Bragg reflections were integrated live using the *EventViewer* program in *ISAW* (Worlton *et al.*, 2004 ▸) during data collection. The quality of the single-crystal sample was determined by examining Bragg peaks in the live data, in a matter of minutes. A single crystal without splitting or multiple Bragg peaks was selected for the study.

Using the strong Bragg peaks, the reduced cell (Niggli cell) and orientation in the instrument, which together define the UB matrix, were determined using a fast Fourier transform (FFT) algorithm and least-squares refined. This UB matrix was then used to index the found Bragg peaks within the specified tolerance of ±0.1 Å^−1^ deviation from individual reciprocal lattice units. As the unit cell is of hexagonal primitive (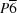) symmetry, no further cell transformation was necessary. The UB matrix was used to plan the experiment using the *Crystal Plan* software (Zikovsky *et al.*, 2011 ▸), which maximizes the coverage of reciprocal space by optimizing the crystal orientations with a genetic algorithm. The data were collected using 11 goniometer settings for approximately 11 h each. A three-dimensional volume of reciprocal space was collected within the wavelength band at each orientation. All orientations combined cover the volume of reciprocal space to be analyzed.

Neutron events, which were detected in detector space (*x*, *y* and TOF), were mapped to three-dimensional reciprocal space. The integration algorithm first found strong Bragg peaks and indexed them using the UB matrix. The single-crystal Bragg peaks were integrated by defining the shape of the integration domain in reciprocal space as a sphere around the center of the Bragg peak (Schultz *et al.*, 2014 ▸). Integration was performed by summing all neutron events inside a chosen radius (in Å^−1^) around each peak center point in reciprocal space. Since the error associated with each event is assumed to be random and independent (Poisson distribution), the errors were summed in quadrature. If the chosen radius resulted in an integration volume that was either partially or entirely off the detector edge, the peak was discarded. The background was estimated by defining a second integration volume, a shell of a specified thickness around the peak, and subtracted.

Using the statistics *versus* integration radius utility written by Schultz *et al.* (2014 ▸) and the β-NaLaF_4_ data, the best radius for integration in reciprocal space was between 0.13 and 0.16 Å^−1^, based on the number of peaks integrated and their 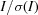 ratios. The integrated intensities were corrected for the wavelength dependence of the incident spectrum, detector efficiency, sample absorption and the Lorentz factor, as described by Schultz *et al.* (2014 ▸). The processed intensities (structure factors) of the indexed peaks were used for structure refinement. In §3, the values were used for comparison with the new general correction protocol, described in §2.3.

The diffuse scattering data were then treated using the standard procedure. The raw event data of each orientation were processed using the *MANTID* (*Manipulation and Analysis Toolkit for Instrument Data*) (Arnold *et al.*, 2014 ▸) software. A geometric detector calibration was applied to correct detector positions relative to the sample and incident beam. The detector spatial distortion was taken into account through calibration at the detector firmware level (Riedel *et al.*, 2015 ▸). The number of events collected per orientation varies according to the neutron flux and the exposure time. In order to combine the data from multiple orientations, the integrated raw events were normalized by the associated beam monitor counts after absorption, spectrum/detector dependent and Lorentz corrections had been applied. The result was a series of individual corrected and normalized data sets representing different volumetric regions of reciprocal space.

**Figure 1:**
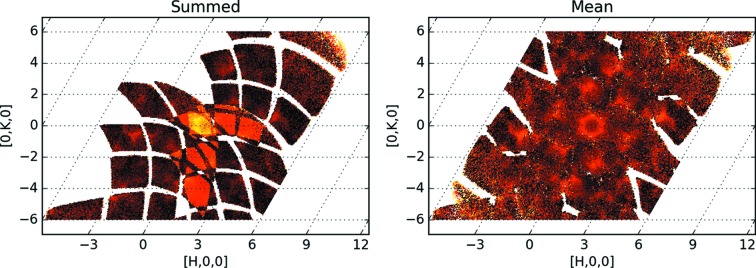
Left: a two-dimensional slice (*L* = −4.5) showing diffuse scattering data from three different sample orientations that were processed the same way as the Bragg scattering, then added together. The scattering cross section cannot simply be added; the increased intensity in the overlap region indicates that an averaging protocol is required. Right: arithmetic mean of contributions from 11 orientations with application of a 180° rotation around the *L* direction.

A reciprocal space reconstruction was attempted by combining the processed data sets. Simply adding three data sets increases the intensity in the overlapping regions, as can be seen in Fig. 1 ▸ (left). Since combining orientations of inherently low statistics (diffuse data) relative to Bragg diffraction is the goal, the non-additive nature of the neutron differential cross section 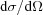 must explicitly be taken into account. This is done by averaging the cross section at every reciprocal space point. Adding the processed data sets and applying the arithmetic mean, or an unweighted mathematical average, by dividing the sum of data per bin (= grid point) by the number of data sets contributing per bin, results in more coverage but does not improve the data (see Fig. 1 ▸, right). Visually, the graininess of the figure does not decrease. Data sets obtained by applying symmetry operations can be treated the same way. Arithmetic averaging is sufficient when all regions of reciprocal space are measured with the same statistical weight.

**Figure 2:**
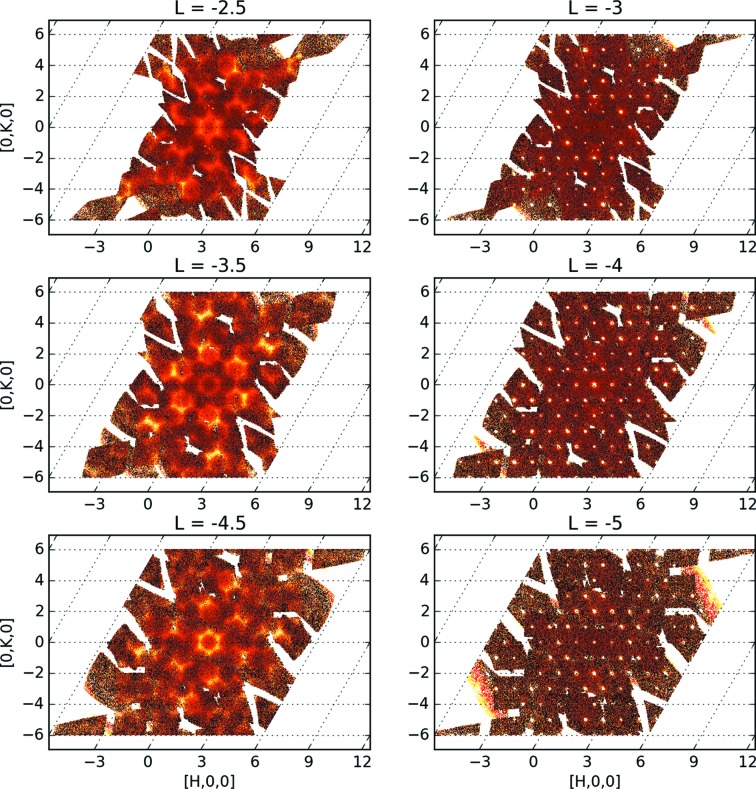
Several two-dimensional slices (*L* = −2.5, −3.0,…, −5.0) showing diffuse and Bragg scattering. Data from 11 different sample orientations were processed using the new protocol described in the text. The smooth transitions in the overlap regions are proof that the statistical weight of the measurement has been correctly taken into account.

Combining 11 orientations of diffuse TOF diffraction data by arithmetic averaging is depicted in Fig. 1 ▸ (right). Welberry *et al.* (2005 ▸) show that the approach works well for good quality data. However, simple averaging of good quality and noisy measurements yields noisy data. The expectation from a physics point of view is that combining a statistically improved with a poor measurement should improve the overall data quality. If the data quality does not improve, regions where quality is poor are usually discarded. Multiple measurements with lower statistics can be useful for data analysis, if the data are treated properly (compare Fig. 1 ▸, right, and Fig. 2, bottom left). Here we present an approach to take into account the statistical weight of the measured data at varying neutron flux.

### 2.3. New data processing approach for diffuse scattering   

To correctly account for the statistical weight of the data, we begin with the definition of the differential scattering cross section (Lovesey, 1984 ▸): 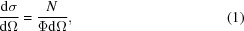
where *N* is the number of scattered neutrons per unit time in an infinitesimal volume (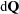) of reciprocal space, around a momentum transfer 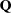, divided by the incident flux (Φ) and the solid angle of the detector (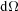). When using multiple detectors, multiple experimental configurations (sample orientations measured for different times or different wavelengths) or polychromatic incident beams, the previous equation needs to be rewritten as 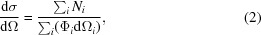
where the summation occurs over all detectors and configurations that contribute to the scattering in the reciprocal space volume element 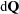. For a given detector *i*, with solid angle 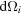, counting 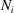 neutrons in such a region, the flux 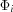 is only that part of the incident beam that can contribute to the scattering around 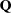. This is especially important for polychromatic beams.

While counting all neutrons that scatter in a certain region of the reciprocal space is straightforward, the calculation of the flux that contributes to that particular scattering is not so obvious. However, as will be shown, this quantity can easily be measured. Equation (2) is applied to an incoherent scatterer, such as niobium doped vanadium. The differential scattering cross section for this material is constant, since the scattering is isotropic:
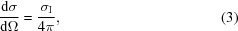
where 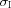 is the total incoherent scattering cross section. If the measurement for the vanadium sample occurs in the same conditions (sample orientation and incident flux) as that for the sample of interest, then 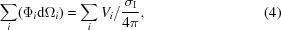
where 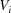 are the neutron counts from vanadium. The sum yields
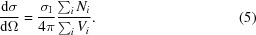



The 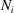 and 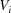 values should be corrected to account for sample and vanadium absorption.

For a single contribution to a particular point in reciprocal space, this is equivalent to the protocol described by Howe *et al.* (1989 ▸) and used in the software for data processing at the SXD instrument at ISIS (Keen & Nield, 1996 ▸). In these previous papers, the authors divide the scattering intensity from the sample by the scattering intensity from the vanadium (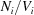 in our notation), then apply some corrections to account for inelasticity, multiple scattering *etc*. In the case of multiple contributions, or multiple symmetry operations (Welberry *et al.*, 2005 ▸), their protocol uses a simple arithmetic mean of various 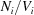 contributions. However, a weighted average should be used instead.

We can rewrite equation (5) as 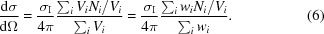



The weight in this case is the number of counts from the incoherent scatterer, in exactly the same region of the reciprocal space, 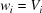. The simple arithmetic mean is a particular case where 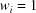 or all 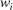 contributions are equal.

As stated earlier, the measurement of vanadium has to occur in the same conditions (sample orientation and incident flux) as the measurement of the sample of interest. This does not mean that the vanadium is not isotropic; it is just the simplest way to correctly keep track of the weights in equation (6). If we assign the same unit cell and orientation to the vanadium as for the sample, the same detectors will contribute with the same incident fluxes for sample and vanadium to each individual region in the reciprocal space.

The same reasoning can be used for symmetrization. Symmetry operations can be applied to the data, as long as the same symmetry operations are applied to the incoherent scattering data, to correctly count the weight at each point in the reciprocal space. The results (11 different orientations and a 180° rotation around the 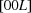 direction) are shown in Fig. 2 ▸. The symmetrized data are considered new data sets, so normalization is calculated independently for each rotation, and symmetrized data and normalizations are added to the original ones, before performing the division. Contrary to the procedure used for Fig. 1 ▸, the overlap region has now been correctly taken into account. This general protocol works for all regions of reciprocal space including both Bragg and diffuse scattering.

Moreover, by correctly accounting for the statistical weights in the overlap region (Fig. 2 ▸) we intrinsically apply both Lorentz and spectrum corrections, as noted by Mayers (1984 ▸). To prove that this is indeed the case, the way these corrections are calculated must be revisited. The coherent scattering differential cross section is related to the unit-cell structure factor 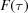 by (Lovesey, 1984 ▸) 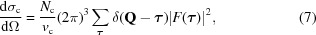
where 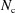 is the number of coherent scatterers, 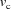 is the unit-cell volume of the scattering crystal, 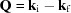 is the momentum transfer to the sample and 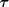 is a reciprocal lattice vector. To obtain the number of scattered neutrons from a particular Bragg peak, we integrate over the solid angle of the detector and multiply by the incident integrated flux as follows: 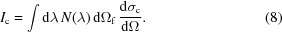
In structure refinements, 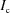 is measured by counting all scattered neutrons in a particular region of reciprocal space, in our case a sphere (but not necessarily).

Given the definition of wavelength, λ, in terms of momentum, *k*, it follows that 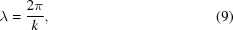


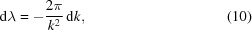


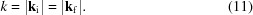



Following the convention that the incident beam is along the 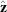 direction and 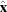 is in the horizontal plane, perpendicular to the incident beam,
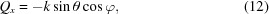


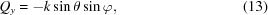


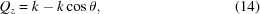
where θ is the conventional polar angle of a spherical coordinate system (not the crystallographic 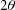 angle) and φ is the azimuthal angle.

The Jacobian for the transformation from 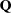 to spherical coordinates of 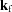 is given by
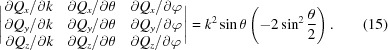



Simplifying of the Jacobian yields for the integration volume element 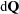


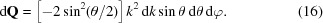
The last three terms on the right hand side represent the scattering solid angle 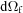.

Using the transformation to spherical coordinates, the intensity for a Bragg peak is given by 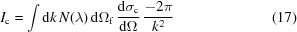


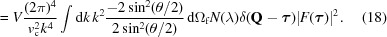



Applying equation (16), and integrating the δ function, yields
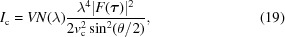
where the sample volume is 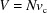.

The structure factor is then related to the integrated intensity by
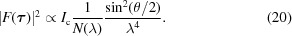
The first fraction represents the spectrum correction; the last is the Lorentz correction and takes into account the amount of time a given reflection remains in the diffraction condition (Buras & Gerward, 1975 ▸). Note that in common crystallographic convention the scattering angle is called 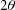, so the Lorentz correction appears as 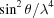. The Lorentz correction has a different form if the experiment is performed on a monochromatic incident beam and the integrated intensity is measured by rocking the crystal.

Similarly, the intensity for an incoherent scattering process is calculated as
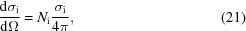
where 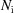 is the number of incoherent scatterers, with a total incoherent scattering cross section 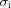.

The corresponding incoherent integrated intensity is given by
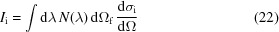


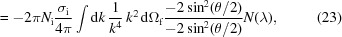


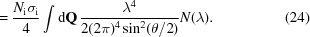



If the data are integrated over a small volume, it can be assumed that λ and θ are approximately constant and can be taken outside of the integral together with 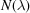. The integral of 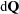 remains, which is the integration volume in reciprocal space and may be chosen as a user-defined constant 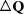.

Following the considerations for the coherent intensity, the incoherent scattering intensity is given similarly by 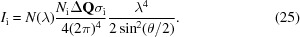



Given that the integration is defined to be over the same small volume for the Bragg peak and the incoherent scattering [equation (25)], the flux term and Lorentz factor are identical, yielding
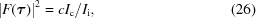
where *c* is a constant that is wavelength and detector independent.

**Figure 3:**
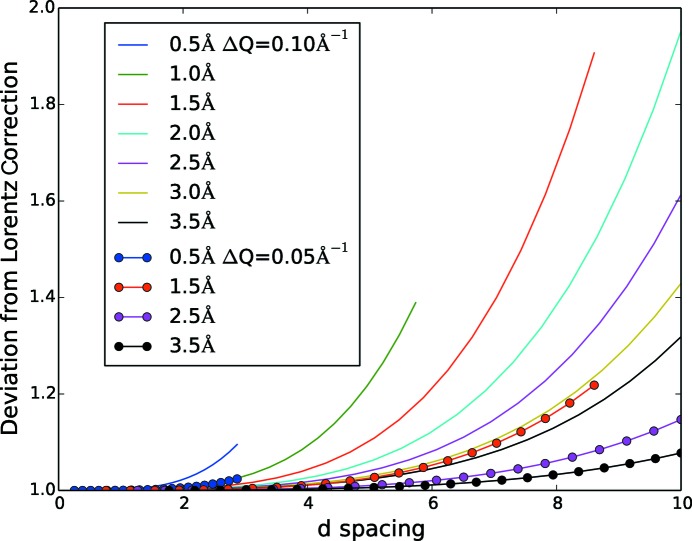
The deviation from the Lorentz correction is shown as a function of *d* spacing for wavelengths in the flux range of 0.5–3.5 Å^−1^ for two different box sizes, 0.10 and 0.05 Å^−1^. Larger *d* spacing and a smaller integration box will result in a smaller deviation from the Lorentz correction.

The ratios of the integral form of the Lorentz correction, equation (24), and the analytical form, equation (25), for selected incident neutron wavelengths and two integration box sizes (0.10 and 0.05 Å^−1^) were calculated and compared in order to estimate the quality of a constant λ and θ approximation for a defined integration region, 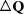, as shown in Fig. 3 ▸. For this comparison we assume that the neutron flux is independent of wavelength for the integration volume. It is apparent that the deviation from the ideal case (ratio = 1) increases with increasing *d* spacing for all wavelengths. The increase is faster for shorter wavelengths. The deviation is more pronounced for the larger, 0.10 Å^−1^, integration box than the smaller, 0.05 Å^−1^, box. This is an expected effect, since an integration region in reciprocal space represents a larger region in detector space at low scattering angles.

**Figure 4:**
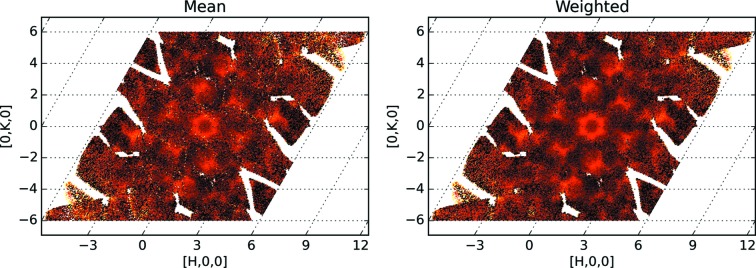
Comparing the old, arithmetic mean protocol (left hand side) and the new, weighted mean protocol (right hand side). The slices at *L* = −4.5 have the same color scale.

We should note that the arithmetic averaging of 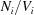 contributions is noisier and has larger error bars than the new weighted mean. A comparison of intensities from Figs. 1 ▸ and 2 ▸ is shown in Fig. 4 ▸.

**Figure 5:**
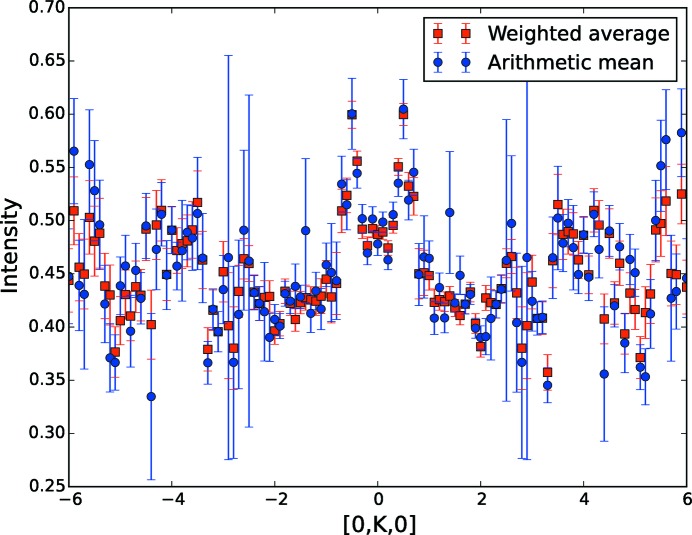
Comparing the old, arithmetic mean protocol (blue circles) and the new, weighted mean protocol (red squares). The cut along the [0*K*0] direction is taken at *H* = 0, *L* = −4.5. The new method yields less noisy data and smaller error bars.

An even more convincing proof is obtained by performing a one-dimensional cut, as shown in Fig. 5 ▸. The cuts are along the [0*K*0] direction, at *H* = 0, *L* = −4.5. The arithmetic mean approach produces data that are less regular and have larger error bars than the proposed weighted mean protocol.

The smaller error resulting from the new protocol can be shown analytically. If we assume the vanadium is measured for a much longer time than the sample (as is most often the case), this implies that the resulting incoherent scattering measurement error is negligible in comparison to that of the coherent scattering. Additionally, in the limit of very good statistics for coherent scattering measurements, all 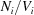 values approach a single constant *I*. The ratio of scattering cross sections from the two methods is
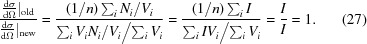



Using Poisson statistics, the error bars for 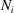 are equal to 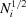. The ratio of the variances for the scattering differential cross sections, 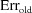 and 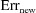, is then given by adding in quadrature the errors from each contribution: 
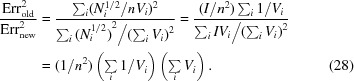



Since 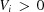, we can use the Cauchy–Schwartz inequality (Cauchy, 2015 ▸) on the previous equation to yield
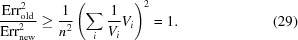



Equality is achieved only when the weights 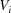 are equal. The error bars for the new method are therefore smaller.

### 2.4. Experimental testing of the new data processing protocol   

A direct comparison between the integration and correction methods described in §§[Sec sec2.2]2.2 and [Sec sec2.3]2.3 is impossible for diffuse scattering data, since the integration area is continuous, although it may be done for Bragg scattering. Two integrated data sets of β-NaLaF_4_ were used for benchmarking the original and new data processing methods through structure refinement: the first from the original post-integration Bragg-only correction method and the second from the new comprehensive reciprocal space correction pre-integration method. For post integration, sample absorption, spectrum and Lorentz corrections were calculated at peak centers and applied to the integrated intensities. Bragg peaks measured at different sample orientations are considered separately, even if they correspond to the same position in the reciprocal space.

In considering the normalization of diffuse scattering data measured on TOPAZ, other corrections including multiple scattering, Debye–Waller factor and any inelasticity effects are negligible and can be ignored. However, it is worth noting that the inelasticity effect is dependent on the ratio of scattered to incident flight paths (Mayers, 1984 ▸), which is insignificant for the TOPAZ instrument, but might be important for other instruments.

Using the same processing protocol for the Bragg data as for diffuse scattering data, the raw data were binned on a regular grid in the *HKL* space. The spacing of the grid was chosen in such a way that the expected Bragg peaks were in the center of each *HKL* box and were completely contained inside individual bins. After the corrections had been applied to all reciprocal space the intensity contained in such a box was considered the integrated intensity of a Bragg peak. The average intensities of the surrounding boxes were calculated as background and subtracted from the signal.

In order to test the proposed procedure with a conventional structure refinement, the Bragg peaks of each orientation were corrected and integrated individually and not combined as has been done to visualize the diffuse scattering. The same reciprocal space point of the sample can be measured in different sample orientations on a different detector and/or at a different neutron wavelength. For every sample orientation we scaled the vanadium data to the same time-integrated incident flux. We associated the same lattice parameters and sample orientation with vanadium as for the corresponding β-NaLaF_4_ measurements. This ensures correction with incoherent scattering at the same position in reciprocal space of the sample with the same statistical weight.

The scattering from vanadium has the same energy dependent spectrum in each detector pixel, up to a pixel dependent multiplicative constant (the efficiency of the detector). This constant varies within 6% of the average for each detector module. However, detector pixels are located at different distances from the sample, so neutrons with the same energy arrive at different detectors at different times; for simplicity all neutron flight times were converted to momentum. Any quantity that depends on neutron velocity could have been selected, such as wavelength, but a constant grid in wavelength results in a logarithmic grid when transformed to the reciprocal space of the sample. A linear grid in momentum space yields a linear grid in momentum transfer.

From equation (26), a low neutron count for vanadium will produce large errors. To decrease background, and avoid regions with a low number of counts, both the coherent and incoherent data were cropped to lie in the [1.85, 9.5] Å^−1^ interval. This also eliminates detected events due to the very high energy neutrons associated with the prompt pulse (Carpenter & Yelon, 1986 ▸). To increase statistics, the momentum dependence of vanadium scattering in detector pixels was averaged with similar energy response. The data were binned using a 0.01 Å^−1^ interval, as the averaging cannot be done in event mode. The bin width was chosen so that it is small enough to be representative of a continuous spectrum but within computational limits for processing the large number of events (20 billion). The binned data were smoothed over each of the 256 by 256 pixels per detector and this average value was expanded over all pixels in each detector. The average values of each detector pixel were multiplied by the integrated value of the detector counts over the momentum range.

## 3. Results and discussion   

The integrated intensities from the standard workflow and new protocol were compared to determine the linearity of their correlation. The intensity statistics as a function of spherical integration radius indicated an optimal integration radius of approximately 0.14 Å^−1^ in 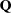 space (§2.2). Integration radii from 0.13 to 0.16 Å^−1^ in 0.005 Å^−1^ increments were tested.

A grid commensurate with the lattice was chosen so that all the Bragg peaks were in the same position inside corresponding *HKL* boxes and to reduce the number of computations. To integrate approximately the same volume in *HKL* space as is used for spherical integration, the box size was defined as a cube of 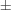
0.1 reciprocal lattice units in each direction.

**Figure 6:**
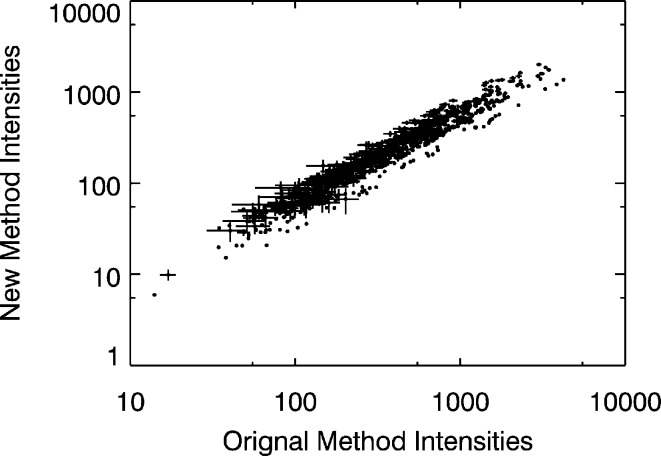
Log–log plot of Bragg peak intensities corrected using the standard processing workflow with spherical integration *versus* the new box integration combined with incoherent scattering normalization.

The same peaks as integrated with the spherical integration method were used in the new integration procedure. Peaks with 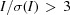 were used for structure refinement. Outlier reflections, where 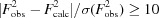, were excluded from the final refinement. The integrated intensities using the original workflow and new protocol were compared. The correlation between the intensities of the two methods calculated as the sample covariance divided by the product of each data set’s standard deviation is high, 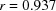 (R Development Core Team, 2008 ▸), and the residuals are randomly distributed about 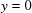, indicating a high positive linear correlation between the two sets of intensities. The error bars in Fig. 6 ▸ show the standard deviations in both methods, which are comparable.

Full anisotropic refinements, including a secondary extinction correction, were performed with the *GSAS* (Larson & Von Dreele, 2000 ▸) program, using the *EXPGUI* (Toby, 2001 ▸) graphical interface, on structure factors of β-NaLaF_4_ obtained from both methods. A summary of the refinement statistics is given in Table 1 ▸.

The refinement results show that the Bragg structure statistics are similar for both integration methods, with 13 reflections rejected in the final round of refinement for the box method. The different shape of the integration and background regions could explain more reflections being accepted for the sphere integration.

**Figure 7:**
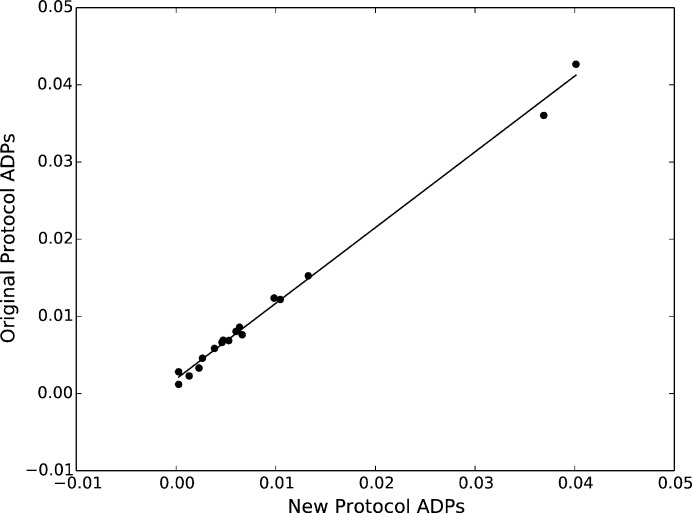
Comparison of anisotropic displacement parameters (slope = 0.98) obtained by *GSAS* refinement for the two methods described in the text.

In the case of major structural differences or refinement issues, the anisotropic displacement parameters (ADPs) would be uncorrelated. However, the results shown in Fig. 7 ▸ are highly correlated (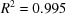). The *F* statistic is 3465 with a corresponding *p* value of 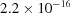, indicating that there is excellent agreement between structural refinement results from sphere and grid integration and the null hypothesis that there is no linear correlation between the data sets should be rejected (R Development Core Team, 2008 ▸).

An X-ray data set was collected as a basis for comparison with the neutron data using a crystal from the same batch [see supporting information; refinement performed using *SHELX97* (Sheldrick, 2008 ▸)]. The ADPs of both neutron integration methods were highly correlated to the X-ray ADPs; 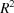 was approximately 0.88 for spherical integration and 0.85 for the new grid integration (R Development Core Team, 2008 ▸). The slightly lower correlation of ADPs between the X-ray and neutron experiments can be explained by the difference in scattering cross sections measured by different methods. These Bragg results demonstrate the validity of the proposed method for the reduction of diffuse intensity data for local structural analysis.

**Figure 8:**
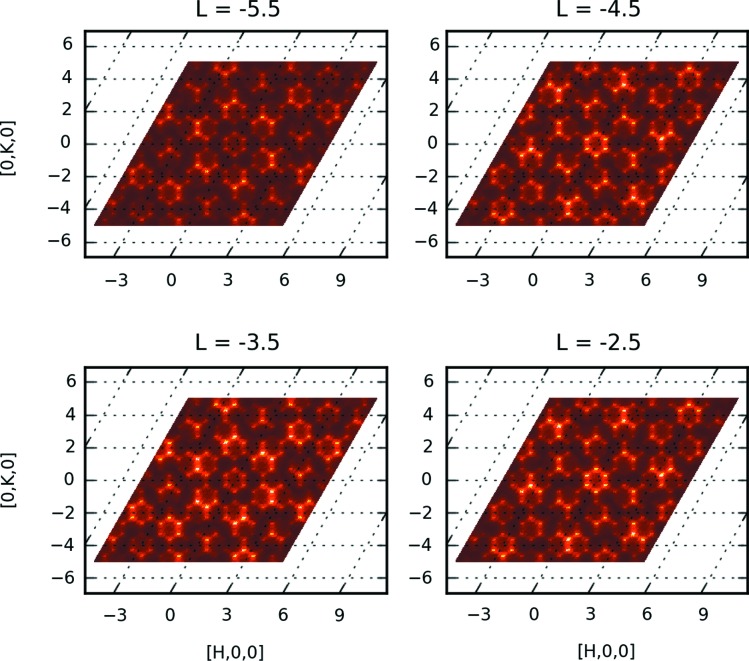
Simulated diffuse neutron scattering in β-NaLaF_4_. The calculation was performed on the same grid as the measured intensities, for layers at *L* = −2.5, −3.5, −4.5 and −5.5. Layers separated by 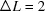 show similar scattering patterns.

As a proof of principle the new normalization protocol was then used to integrate the diffuse intensities of four layers (*L* = −2.5, −3.5, −4.5 and −5.5). A supercell was defined by doubling the *c* axis, resulting in integer values for *L* for computational modeling (Chodkiewicz *et al.*, 2010 ▸). We masked the aluminium powder rings from the sample pin corresponding to the 111, 200, 220 and 311 reflections using the *MANTID* (Arnold *et al.*, 2014 ▸) software. The data sets were binned with a step size 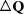 = 0.1 in reciprocal lattice units. In principle, a much smaller step size may be needed to extract the diffuse intensities and their associated standard uncertainties for quantitative computational modeling (Michels-Clark *et al.*, 2013 ▸) as the grid definition is not restricted by the necessity of integer *HKL* values. Intensities were calculated on the same grid as the experimental data using a Monte Carlo model of the local structure, as described by Aebischer *et al.* (2006 ▸), in a diffuse scattering modeling software currently under development, *ZODS* (*Zurich Oak Ridge Disorder Simulations*; Chodkiewicz *et al.*, 2010 ▸). The four simulated diffuse layers are shown in Fig. 8 ▸. The measured data in Fig. 2 ▸ are in good agreement with the simulation. Comparing with X-ray data from Aebischer *et al.* (2006 ▸) the complementarity of scattering length differences between X-rays and neutrons is evident and can be used as a seed for comprehensive analysis development.

## 4. Summary   

In this work, we present a comprehensive correction and normalization protocol for total scattering data. The ratio of integrated intensities, over the same small volume in the reciprocal space, for the coherent and incoherent scattering intensity is shown to implicitly account for the flux term and Lorentz contributions. *GSAS* Bragg refinements of structure factors from the new and old methods produce highly comparable structural results, with low *R* values and highly correlated ADPs. This enables the use of corrected diffuse structure factor data as input to local structure modeling software. It is imperative that all coherent data and all incoherent data are processed comprehensively, each of them summed separately for different orientations, and normalized only at the end. The same principle applies to symmetrization: symmetrize data, add it to the original data, symmetrize normalization and add it to the original normalization, then divide the sums. This way the statistical weights of the measurements are preserved. The methods presented here work equally well on event or histogrammed data, and provide a first step for automated processing of total scattering neutron TOF single-crystal diffraction data.

## Supplementary Material

Comparisons of X-ray data sets and neutron data. DOI: 10.1107/S1600576716001369/fs5119sup1.pdf

## Figures and Tables

**Table 1:** *GSAS* Bragg refinement statistics from spherical integration in the original workflow and new protocol grid integration The spherical integration radius for the old method is 0.14 Å^−1^. The box size for the new method is 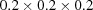 reciprocal lattice units. Peaks are rejected if 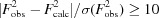.

Method	Peaks	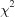	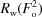	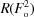	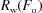	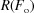	Extinction	Rejected
Sphere	938	3.434	0.102	0.084	0.051	0.048	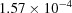	0
Grid	929	6.063	0.075	0.087	0.038	0.051	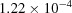	13
