# Expanding Lorentz and spectrum corrections to large volumes of reciprocal space for single-crystal time-of-flight neutron diffraction. Corrigendum

**DOI:** 10.1107/S1600576717006781

**Published:** 2017-09-25

**Authors:** Tara M. Michels-Clark, Andrei T. Savici, Vickie E. Lynch, Xiaoping Wang, Michal Chodkiewicz, Thomas Weber, Hans-Beat Bürgi, Christina M. Hoffmann

**Affiliations:** a Lawrence Berkeley National Laboratory, Berkeley, CA 94720, USA; b Neutron Sciences Directorate, ORNL, PO Box 2008 – MS 6475, Oak Ridge, TN 37831-6475, USA; c Department of Chemistry, Warsaw University, Pasteura 1, Warsaw 02-093, Poland; d Laboratory of Crystallography, ETH Zürich, Wolfgang-Pauli-Strasse 10, HCI G505, CH-8093 Zürich, Switzerland; e Department of Chemistry and Biochemistry, Universität Bern, Freiestrasse 3, CH-3012 Bern, Switzerland

**Keywords:** modulated diffuse scattering, local structure modeling, Lorentz and spectrum corrections, single-crystal time-of-flight neutron diffraction

## Abstract

The author list of the article by Michels-Clark *et al.* [*J. Appl. Cryst.* (2016), **49**, 497–506] is amended with the addition of Michal Chodkiewicz, Thomas Weber and Hans-Beat Bürgi. The complete list of authors is Tara Michels-Clark, Andrei Savici, Vickie Lynch, Xiaoping Wang, Michal Chodkiewicz, Thomas Weber, Hans-Beat Bürgi and Christina Hoffmann.

## Abstract 2

Corrigendum to *J. Appl. Cryst.* (2016), **49**, 497–506: addition of co-authors.

The author list of the publication by Michels-Clark *et al.* (2016 ▸) is amended, with Michal Chodkiewicz, Warsaw University, Thomas Weber, ETH Zürich, and Hans-Beat Bürgi, Universities of Bern and Zürich, as full-fledged co-authors. Tara Michels-Clark, Andrei Savici, Vickie Lynch, Xiaoping Wang and Christina Hoffmann apologize for the omissions.

The contributions of the individual authors to the article are as follows:

Tara Michels-Clark, Vickie Lynch, Andrei Savici, Xiaoping Wang and Christina Hoffmann prepared the manuscript, compared different correction algorithms for the diffuse scattering neutron diffraction data, and analyzed and developed the data processing workflow. The mathematical treatment of the Lorentz-correction and statistical weighting scheme was devised by Andrei Savici and Tara Michels-Clark.

The neutron data were obtained in a joint experiment between the ORNL and Zürich groups. Neutron single-crystal diffraction data from the SNS TOPAZ diffractometer were collected and refined by Tara Michels-Clark, Christina Hoffmann and Xiaoping Wang. Complementary X-ray data at 100 K were measured and refined by Tara Michels-Clark and Christina Hoffmann. Tara Michels-Clark statistically compared the Bragg data obtained by different processing protocols.

Tara Michels-Clark analyzed the diffuse data using the Monte Carlo crystal builder and intensity calculations in *ZODS*. Michal Chodkiewicz, Thomas Weber and Hans-Beat Bürgi developed the *ZODS* software for model building and refinement. Hans-Beat Bürgi co-mentored Tara Michels-Clark’s PhD thesis with Christina Hoffmann.
