# Supplementary material for: Expanding Lorentz and spectrum corrections to large volumes of reciprocal space for single-crystal time-of-flight neutron diffraction
Source: J Appl Crystallogr. 2016 Mar 1;49(Pt 2):497–506. doi: 10.1107/S1600576716001369 (PMC4815874; doi:10.1107/S1600576716001369)
Supplement: Supplementary file 1 [file j-49-00497-sup1.pdf]

Supplementary Material

An X-ray data set was collected as a basis of comparison to the neutron data using a crystal from the same batch. A crystal of globular shape with dimensions 0.12x0.1x0.09mm^3 was used in the data collection. The crystal structure has hexagonal symmetry with space group $P\bar{6}$ and was refined with unit cell dimensions a = 6.1520(14)Å, c = 3.8191(9)Å and unit cell volume, V = 125.18(6)Å^3 with density $\rho_{calc} = 4.734$ gcm^{-3}. A Bruker Smart 1 K area detector diffractometer with graphite-monochromated MoK$_\alpha$ radiation and wavelength $\lambda = 0.71013$ Å using 0.3° ω scans and SMART and SAINT software were used. A total of 816 reflections were measured resulting in 239 unique reflections at angle $2\theta_{max} = 56.37^\circ$. An empirical absorption correction with SADABS (ref) was done with μ = 12.848 mm^{-1} and the minimum and maximum transmission were T_{min} = 0.5704 and T_{max} = 0.7457, respectively. A least squares, full-matrix refinement against F2 with SHELX97 (Sheldrick, 2008) using all 816 reflections ($I > 2\sigma(I)$) and 22 parameters resulted in R_1 = 0.0138 ($I > 2\sigma(I)$) and wR_2 = 0.0346 (all data). An inversion twin with the ratio 0.43 was refined. The residual electron density was $+0.43/-0.51$Å^3.

For both neutron integration methods, ADPs were highly correlated to the X-ray ADPs , the correlation coefficient r was approximately 0.938 for spherical integration and 0.922 for Box integration (figure S.1).

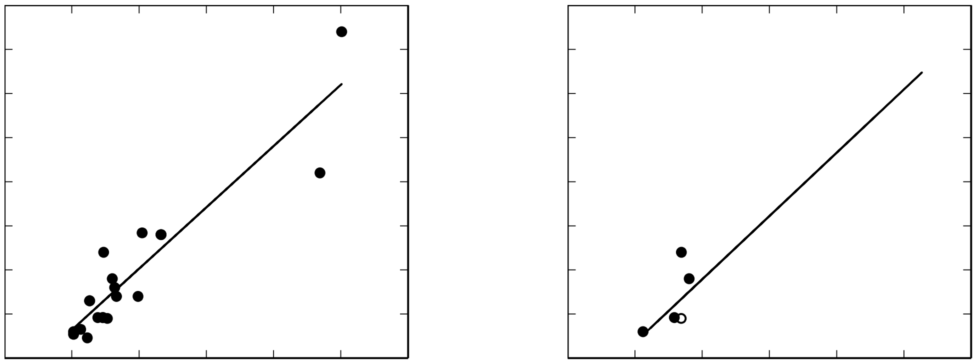

Figure S.1 The correlation between the ADPs of new protocol (left hand side) and original protocol (right hand side) with those from the Bragg X-ray structure.

Sheldrick, G. M. (2008). *Acta Crystallogr. A*, **64**,112-122.
